# Assessing the Impact of a Modified Core Decompression Technique on Early-Stage Avascular Necrosis of the Hip Using Bone Marrow Concentrate Adjuvant Therapy: A Retrospective Study

**DOI:** 10.7759/cureus.69271

**Published:** 2024-09-12

**Authors:** Anant Tiwari, Kamini Khillan, Mayank Poddar, Vivek Ranjan

**Affiliations:** 1 Orthopedics, Sir Ganga Ram Hospital, New Delhi, IND; 2 Pathology, Sir Ganga Ram Hospital, New Delhi, IND; 3 Orthopedics, BLK-Max Super Speciality Hospital, New Delhi, IND

**Keywords:** avascular necrosis, bone decompression, bone marrow concentrate, neck femur, osteonecrosis

## Abstract

Background: Osteonecrosis is characterized by the necrosis of trabecular bone and cells within the femoral head, which often results in the subchondral collapse and deformation of the articulating surface of the head. For the treatment of early stages of this condition, specifically Stage I and Stage II, bone marrow-derived stem cells have been employed effectively for several years. In our approach, we have utilized a modified technique to collect bone marrow aspirate, which has yielded favorable outcomes.

Methods: In our study, we performed surgeries on 32 hips afflicted with early-stage osteonecrosis of the femoral head. Each patient underwent core decompression and the injection of bone marrow concentrate, guided by C-arm imaging in the operating theater. Evaluations were conducted using the Harris Hip Score and the Visual Analogue Scale (VAS), along with radiological assessments to track the progression of osteonecrosis stages before and after the surgical procedure.

Results: The comparison of pre- and post-surgery data, including the Harris Hip Score, VAS, progression of osteonecrosis stages, and radiological findings, revealed significantly positive outcomes. Since May 2013, 32 hips, regardless of the etiology of avascular necrosis (AVN), have been treated with this procedure. Notably, only four patients with bilateral AVN progressed to Stage III in one hip, while the other hip remained stable. In the remaining patients, pain was alleviated, and none progressed to later stages. No complications were observed in this study.

Conclusion: This minimally invasive technique, characterized by its simplicity and lack of associated complications or donor site morbidity, has proven to be an effective joint-preserving surgical intervention for early stages of femoral head osteonecrosis (Stages 1 and 2).

## Introduction

Avascular necrosis (AVN) of the femoral head is characterized by the loss of bone cells within the femoral head, leading to the collapse of its articular cartilage and subsequent damage to the acetabular cartilage. The underlying pathophysiology involves vascular clotting that causes a generalized venous blockage, ultimately disrupting the microcirculation of the femoral head [1]. Initial discomfort in AVN is attributed to elevated intraosseous pressure (pre-collapse: Stages 1 and 2), the discomfort further progresses due to subchondral fractures (Stage 3). In later stages (Stages 4 to 6), discomfort arises from the loss of joint surface congruency [2]. Eventually, the majority of affected hips require total hip arthroplasty [3].

For many years, stem cells derived from bone marrow have been utilized in the early stages of AVN. These stem cells are capable of differentiating into various cell types, aiding in the repair of the femoral head [4]. These mesenchymal cells can survive in the ischemic environment of the femoral head for several months [5].

Bone marrow-derived mesenchymal stem cells (MSCs) are particularly effective in treating conditions like joint aging osteonecrosis and non-union of bones due to their ability to develop into various cell types, along with secreting cytokines and growth factors [6,7]. In 1990, it was proposed that autologous stem cell implantation could be a viable treatment for AVN [8].

The effectiveness of stem cells in treating AVN is partly due to their bone-forming ability and secretion of cytokines and growth factors [9]. After the age of 25, the count of osteoprogenitor cells in the femoral head diminishes, with a more pronounced reduction observed in cases of necrosis. Although there is no conclusive evidence that the number of osteogenic progenitor cells directly correlates with treatment efficacy, patient response appears to be influenced by the total count of injected mononuclear cells (MNCs). Favorable results have been reported with a total count of approximately 30x103 osteoprogenitor cells [10].

Typically, the aspirate contains a small number of mononuclear cells, about 1x10^5^, which can be increased fivefold by centrifugation techniques. Recent studies suggest that a clinically significant mean count is around 25x10^3^ per hip [11].

Connolly et al. were pioneers in developing a method for preparing bone marrow [12]. The most frequently studied techniques for obtaining high yields of osteogenic cells include density gradient centrifugation and cell separator operations.

Sakai et al. proposed that MSCs can be efficiently extracted from bone marrow using a buffy coat extraction method [13]. They further refined this approach by collecting bone marrow in a quadruple top and bottom bag system, followed by centrifugation to concentrate the aspirate. This process is then completed with manual separation [13]. This innovation has contributed significantly to the field of bone marrow stem cell extraction, offering a more effective way to isolate and utilize these cells for therapeutic purposes.

## Materials and methods

This study was reviewed and approved by the Institutional Ethics Committee of Sir Ganga Ram Hospital, New Delhi with the approval number EC/02/22/2001. We developed a simple method for core decompression and the implantation of bone marrow concentrate in early stages (Stage I and II, according to Steinberg Classification [2]) of AVN of the femoral head, designed to stabilize the hip and promote lesion healing. The study included patients with Stage I and II AVN, regardless of etiology.

A total of 18 patients (32 hips), with varying causes of AVN, underwent this procedure. Among them, four had unilateral AVN, and 14 had bilateral AVN. In the operating theater, under standard aseptic precautions, bone marrow was aspirated from the anterior superior iliac spine (ASIS) and iliac tubercle using a heparinized Jamshedi bone biopsy needle. For a single hip, 50 ml of bone marrow was aspirated, and for bilateral procedures, 100 ml was collected. For each hip, a 50 ml syringe was used to aspirate bone marrow, and in bilateral cases, two syringes (totaling 100 ml, 50 ml from each site) were used. The syringes were heparinized to prevent clotting during the aspiration process, ensuring that the bone marrow remained viable for concentration by preventing coagulation. Throughout the procedure, the needle remained in a single fixed position without being repositioned or moved. This aspirated bone marrow was then placed in four-part centrifugation bags by the assisting surgeon and sent to the blood bank for separation of the buffy layer of mononuclear cells under strict aseptic conditions.

A specialized "top-and-bottom" bag system (Fresenius Kabi, Bad Homburg, Germany) featuring outlets at both ends of the collecting bag, was used to collect bone marrow in the operating theater. A citrate-phosphate-dextrose solution in the mother bag was reduced from the standard 63 ml for 450 ml of blood collection to the amount required for the collection of 50 or 100 ml of bone marrow (50 ml for a single hip, 100 ml for bilateral hips).

The volume of the aspirated material was reduced to enhance its stem cell content, selectively retaining nucleated cells such as mononuclear stem cells, monocytes, and lymphocytes, while removing non-nucleated red blood cells and plasma. The "top-and-bottom" bag system facilitated the simultaneous removal of red cells and plasma in a controlled, closed-system process, minimizing contamination risks and ensuring precise final product volume.

The processed bone marrow, concentrated to 20 ml for hip joint injection, was prepared within 30 minutes using a standard blood bank centrifuge (Heraeus Cryofuge 6000i, Thermo Fisher Scientific, Waltham, MA, USA, at 3900 RPM for 10 minutes at 22°C with a g-force of 493). The final concentrated volume from 50 ml of bone marrow for a unilateral procedure yielded approximately 10 ml.

The method we employed for core decompression in our patients with AVN of the femoral head was meticulously planned and executed. The process began with a small, 2.5-inch incision at the lower part of the trochanter on the lateral thigh. After incising the deep fascia, the vastus lateralis was cut at the level of the trochanteric ridge with an L-shaped incision. We then created a single hole using a 4.5 mm drill bit, positioned 10-20 mm below the trochanteric ridge. Through this pilot hole, five tracts were drilled to the four quadrants and the center of the femoral head using very slow reverse drilling under C-arm guidance. This drilling was careful to breach the sclerotic cavity in any quadrant, ensuring all tracts were within 5 mm of the subchondral bone.

The previously prepared bone marrow concentrate, now back in the operating theatre, was meticulously drawn into a 10 ml syringe. We used a Jamshedi needle for the injection, specifically targeting the tract with the sclerotic cavity under C-arm guidance. The injection of the concentrate was performed very slowly to prevent any leakage through the pilot hole. Following the injection, the pilot hole was sealed with bone wax, and the wound was closed without a drain.

Postoperative follow-up for our patients was comprehensive and extensive, spanning over five years. Initial follow-up visits were scheduled at three weeks, six weeks, three months, six months, and one year, with annual check-ups thereafter for five years. Stitch removal occurred at the three-week mark. At each follow-up, patients were evaluated using the Harris Hip Score and the Visual Analogue Scale (VAS). A comparison between the preoperative and postoperative scores was specifically conducted six months after the surgery to assess the effectiveness of the treatment.

## Results

Our study, initiated in May 2013, has yielded significant outcomes in assessing the effectiveness of a specific surgical procedure for AVN. We evaluated the procedure's impact using the Harris Hip Score, VAS, and radiological assessments, focusing on the progression of osteonecrosis stages as shown in Table 1. The study encompassed 18 patients, totaling 32 hips affected by AVN, without restriction to the cause of the condition. Table 1 shows a significant reduction in pain (VAS scores) and improvement in hip function (Harris Hip Scores) across all stages following the surgery. The surgery appears to be most effective in the early stages (I and IIa), with a 100% survival rate, indicating that all treated hips maintained their stage and did not require further intervention. In Stage IIb, the survival rate drops to 90%, suggesting some progression or need for further treatment in 10% of the cases. Stage IIc shows a much lower survival rate of 25%, indicating that the modified technique may be less effective in advanced cases of osteonecrosis.

**Table 1:** Results of the pre- and post-operative examinations and the survival rate according to lesion size and stage (Steinberg classification).

Stage	Total number of hips result N (%)	Pre-operative		Post-operative		Survival (%)
		Visual Analogue Scale	Harris Hip Score	Visual Analogue Scale	Harris Hip Score	
I	8 (25)	3.5	74	1.25	93	100
IIa	10 (31.25)	4.3	69.3	1.5	92	100
IIb	10 (31.25)	5.6	64	1.8	86	90
IIc	4 (12.5)	6.5	62.5	2.75	81	25
Total	32					78.75

Notably, among patients with bilateral AVN, only four experienced progression to Stage III in one hip, while the procedure successfully prevented the advancement in their other hip. Specifically, one hip initially in Stage IIb and three hips in Stage IIc progressed to Stage III. Conversely, in the remaining patient group, we observed complete pain resolution, with no further progression to advanced stages of AVN. Moreover, our study recorded no perioperative or postoperative complications in these cases, underscoring the safety and potential efficacy of the surgical intervention for AVN management.

## Discussion

**Figure 1:**
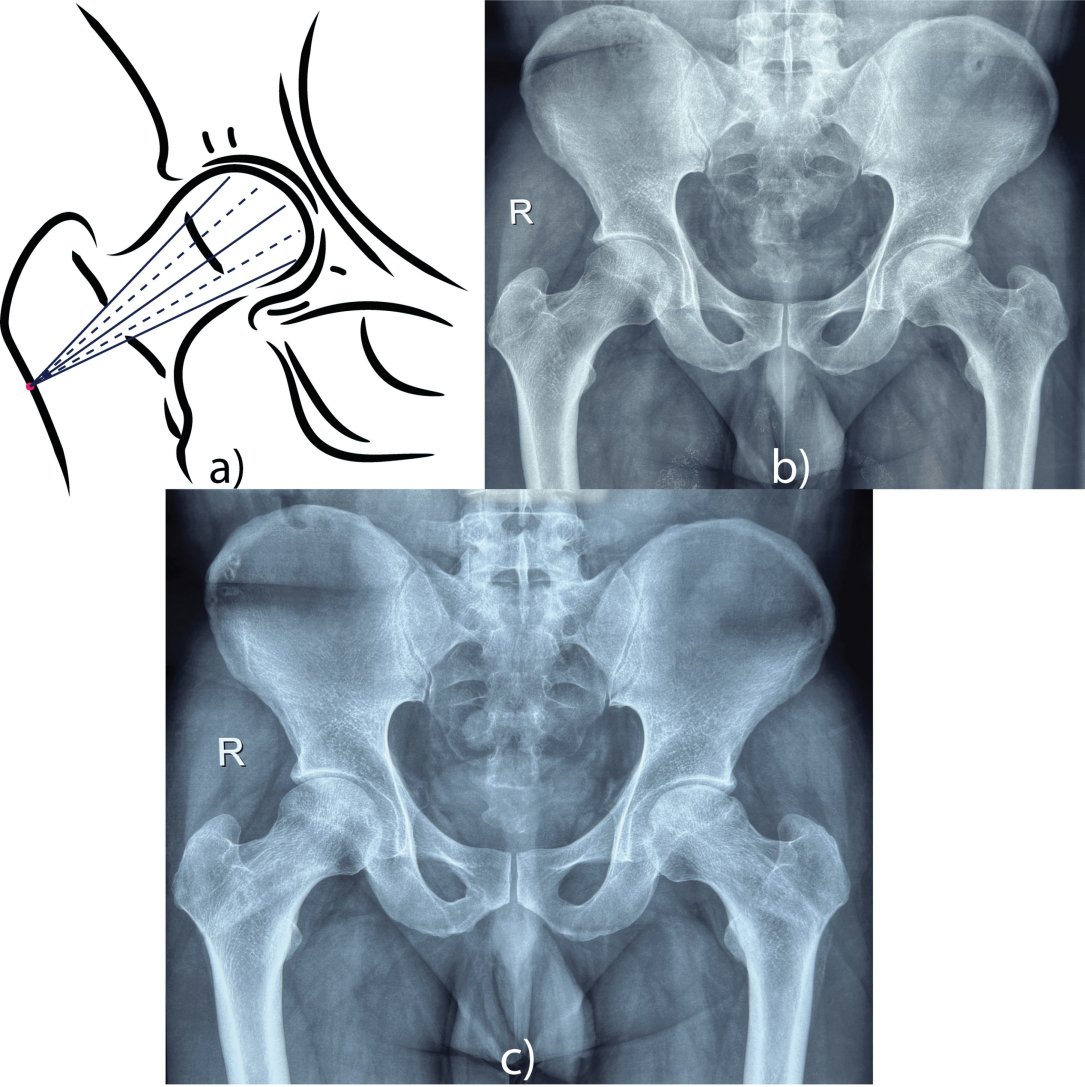
A sequential depiction of the modified core decompression technique used for treating avascular necrosis (AVN) of the femoral head, highlighting both the method and the progressive healing stages. (a) Outlines the surgical method, where a single pilot hole was drilled using a 4.5 mm drill bit into all four quadrants and the center of the femoral head through reverse drilling, following which bone marrow concentrate was inserted into the affected area. The remaining portion of the image contains radiographic images showing the stages of the disease and the healing process. (b) Stage I and Stage II AVN on the right and left sides, respectively, with a sclerotic cavity in the femoral head. (c) Complete healing of the lesion within six months, indicating the effectiveness of the combined surgical and biological approach in restoring femoral head integrity and function.

Figure 1 presents a sequential depiction of the modified core decompression technique used for treating avascular necrosis of the femoral head, highlighting both the method and the progressive healing stages. The upper portion of image (a) outlines the surgical method, where a single pilot hole is drilled using a 4.5 mm drill bit into all four quadrants and the center of the femoral head through reverse drilling. This procedure allows for the insertion of bone marrow concentrate into the affected area. The remaining portion of the image contains radiographic images showing the stages of the disease and the healing process. The first radiograph (b) demonstrates Stages I and II of AVN on the right and left sides respectively with a sclerotic cavity in the femoral head. The final image (c) shows complete healing of the lesion within six months, indicating the effectiveness of the combined surgical and biological approach in restoring femoral head integrity and function.

Osteonecrosis of the femoral head is a significant medical condition, contributing to 5-12% of hip arthroplasties [14]. Each year, an estimated 10,000 to 20,000 new cases of AVN hip are diagnosed [15]. Untreated, this condition can lead to the collapse of the femoral head and severe degeneration of the hip joint, characterized by reduced blood supply to the femoral head, bone cell death, subchondral bone fractures, intense hip pain, and mobility loss [16,17].

The primary treatment goal for AVN, particularly prevalent among individuals in their 20s and 30s, is hip joint preservation. A key strategy involves removing necrotic tissue and replacing it with healthy cells. Several methods have been proposed for AVN treatment, such as osteotomies, total hip arthroplasty, core decompression (with or without bone grafting), and core decompression combined with bone marrow aspiration concentrate. However, these treatments are not universally suitable for all ages and stages of development. In advanced cases, hip replacement is considered, but this is not ideal for younger patients due to the potential need for future surgeries [18].

Our current study focuses on treating early-stage AVN using core decompression paired with bone marrow aspirate concentrate, employing a simplified method involving blood bank facilities. This approach resulted in a 78.75% survival rate without donor site morbidity. While other studies support these findings, our methodology for obtaining bone marrow concentrate is notably more straightforward.

It's observed that AVN femoral heads lack osteoprogenitor cells, leading to less effective outcomes with simple core decompression surgery. Thus, the addition of bone marrow aspirate concentrate or bone grafting is recommended. Pioneering work by Hernigou and Beaujean in 2002 treated over 180 hips with promising results [19]. Zhao et al.'s comparative study between core decompression alone and core decompression with bone marrow aspirate concentrate showed significantly better outcomes in the latter group, with over 90% of early-stage AVN cases exhibiting excellent results as they demonstrated a decrease in osteoblastic activity in necrotic bone with bone marrow concentrate [20].

Recent research indicates that core decompression with bone marrow aspirate concentrate effectively reduces disease progression in pre-collapsed stages of AVN [21-23]. However, this treatment is less effective in advanced AVN cases with head collapse [24]. Other studies have demonstrated that core decompression alone does not significantly heal a necrotic femur head, as confirmed through post-surgery histopathology and MRI [25].

Historically, core decompression with fibula bone grafting (vascularized or non-vascularized) has been employed in early-stage AVN treatment, showing promising results. Yet, this method can lead to various donor site complications, such as infection, pain, nerve palsy, and ankle instability, affecting patient rehabilitation. Vascularized fibula grafting, being more complex and resource-intensive, is not feasible in all medical settings [26-29]. Tantalum rod implantation, while effective, is seldom used due to its cost [30].

The study has certain limitations that should be considered. Firstly, the small sample size of 18 patients (32 hips) reduces the statistical power and generalizability of the findings. The absence of a control group limits the ability to determine if the observed healing is specifically due to the procedure rather than other factors. The procedure's reliance on specific equipment and blood bank facilities may restrict its applicability in other settings without similar resources. Additionally, the study is conducted at a single center, which may introduce bias and affect the generalizability of the findings. These limitations suggest that further research with larger, more diverse populations and longer-term follow-up is necessary to validate these findings and better guide clinical practice.

## Conclusions

Our research, which included a thorough review of various medical websites and literature, revealed no existing publications that describe this straightforward technique for preserving the femoral head in the early stages of avascular necrosis (AVN) or an effective method for acquiring bone marrow concentrate. The method we developed, which involves multiple quadrant decompression and bone marrow concentrate infiltration through a single pilot hole, is simple, safe, and highly replicable. It can be implemented in any healthcare center equipped with a C-arm machine and a blood bank, requiring minimal financial investment. This minimally invasive technique is easy to perform, free from complications and donor site morbidity, and does not lead to significant perioperative issues, making it both a cost-effective and efficient option for treating early-stage AVN.

The outcomes observed in our study were excellent, with successful preservation of hip anatomy, arrest of disease progression, and the potential to avoid premature total hip arthroplasty. The technique also facilitates early postoperative weight-bearing as tolerated, which aids in patient rehabilitation and recovery. Based on our findings, we conclude that this method is an effective joint-preserving surgical intervention for the early stages of osteonecrosis of the femoral head (Stages I and II). Its simplicity, safety, and cost-effectiveness make it a practical and accessible option for a wide range of healthcare settings, offering a valuable alternative to more invasive procedures.
